# Systemic amyloidosis in a patient presenting with myopathy, peripheral oedema and proteinuria

**DOI:** 10.5694/mja2.51667

**Published:** 2022-08-04

**Authors:** Laura Bywater, Anthea C Gist, Rahul G Muthalaly, Joanna Loh, Ian Simpson, Anthony J White, Andy KH Lim

**Affiliations:** ^1^ Monash Health Melbourne VIC; ^2^ Monash University Melbourne VIC

**Keywords:** Bone marrow diseases, Myeloma, Cardiomyopathies, Heart failure, Ventricular dysfunction, Echocardiography, Kidney diseases, Glomerulonephritis

## Clinical record

A 58‐year‐old man initially presented to a private physician with 4–6 weeks of lower limb muscle weakness, an elevated serum creatine kinase level of 344 U/L (reference interval [RI], 40–250 U/L), painful paraesthesia, and weight loss. He was treated with mycophenolate mofetil and prednisolone for suspected idiopathic inflammatory myopathy. Initial investigations did not detect myositis‐specific antibodies or focal muscle oedema on magnetic resonance imaging of his thighs. His symptoms progressed to worsening peripheral oedema, dyspnoea and functional decline, and he was admitted to hospital 4 months after symptom onset. His history included gout, and he was a non‐smoker with moderate alcohol intake.

On admission, he had signs of proximal myopathy and pitting oedema to the thighs, along with non‐tender, non‐pulsatile hepatomegaly and a left lower lobe pneumonia on chest x‐ray. An electrocardiogram showed sinus rhythm, right axis deviation, right bundle branch block, and inferior Q waves. His cardiac troponin I level was elevated at 139 ng/L (RI, < 20 ng/L). He proceeded to have a chest computed tomography (CT) scan and a transthoracic echocardiogram. While showing a parapneumonic collection, the CT scan identified a markedly abnormal left ventricle, which on transthoracic echocardiography appeared as markedly increased left and right ventricular wall thickness with reduced contraction in all regions except the apex, along with severely reduced early diastolic mitral annular velocities and bi‐atrial enlargement (Box 1).

Box 1 **Abnormal cardiac imaging**

**Figure:**
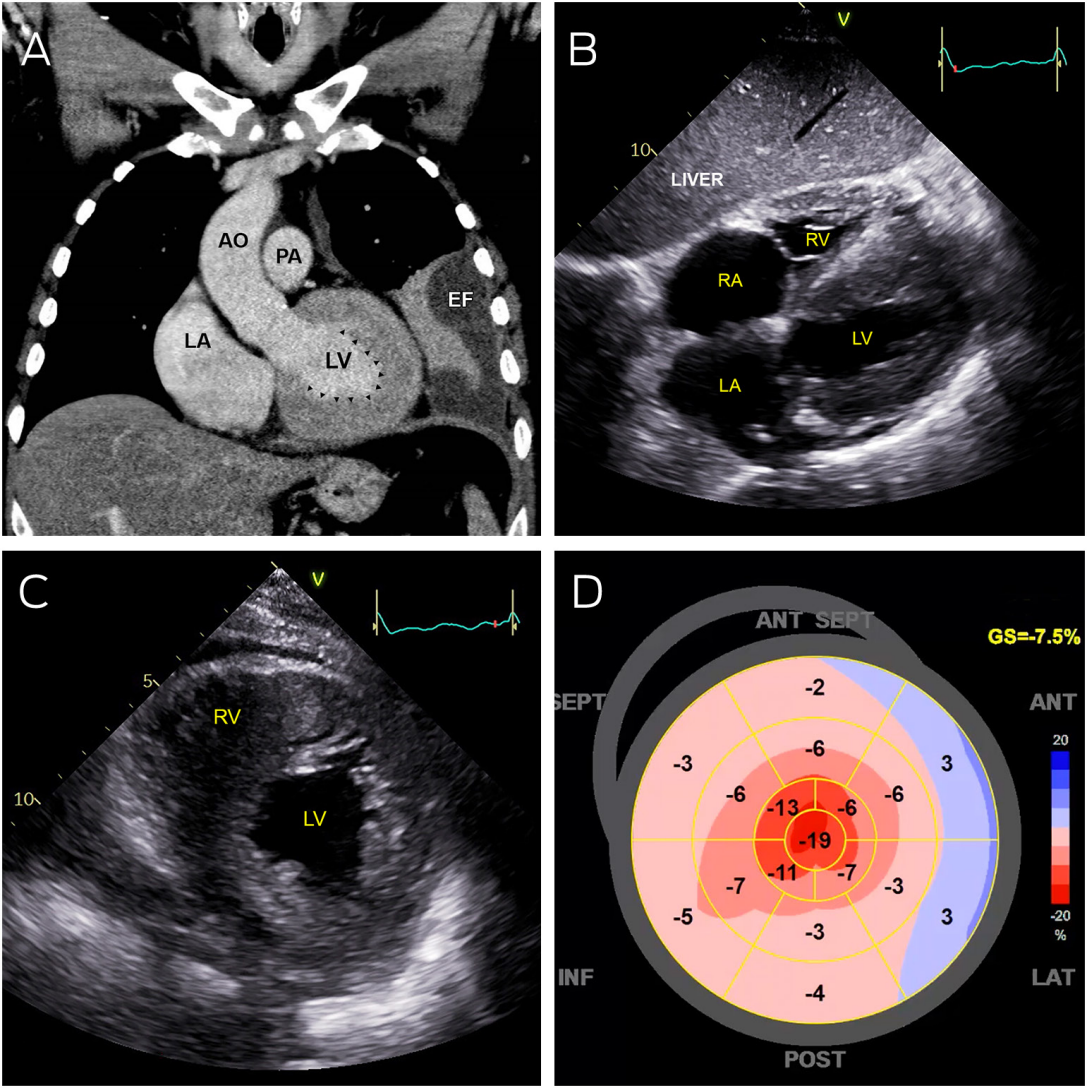
AO = aorta; EF = pleural effusion; LA = left atrium; LV = left ventricle; PA = pulmonary artery; RA = right atrium; RV = right ventricle. **A:** Coronal computed tomography showing severe left ventricular thickening without dilatation (arrowheads). **B:** Transthoracic echocardiography (subcostal view) showing severely thickened left and right ventricular walls, with severe bi‐atrial dilation. **C:** Transthoracic echocardiography (parasternal short axis view) showing severe increased left and right ventricular wall thickness. **D:** Strain map of the left ventricle showing systolic shortening of different walls of the left ventricle in a 2‐dimensional flattened image. Areas of bright red represent normal contraction while pink and blue areas represent hypocontractility. This strain map demonstrates apical preservation of contraction referred to as the cherry‐on‐top sign.

Liver function testing showed an albumin level of 24 g/L (RI, 32–47 g/L), bilirubin 13 μmol/L (RI, < 20 μmol/L), alkaline phosphatase 126 U/L (RI, 30–110 U/L), γ‐glutamyltransferase 208 U/L (RI, 5–50 U/L), and alanine aminotransferase 43 U/L (RI, 5–40 U/L). Abdominal ultrasound confirmed hepatomegaly. Renal function and ultrasound were normal, but urinalysis showed a leucocyte count of 15 × 10^6^/L (RI, < 10 × 10^6^/L), an erythrocyte count of 43 × 10^6^/L (RI, < 10 × 10^6^/L), and a urine protein–creatinine ratio of 0.37 g/mmol (RI, < 0.03 g/mmol). Autoimmune and vasculitis markers were negative, and no evidence of other infection was found. There was sensorimotor peripheral neuropathy and myopathic features on nerve conduction and electromyography studies but no evidence of fibre necrosis.

In the absence of aortic stenosis or history of hypertension, the differential diagnosis of ventricular wall thickening included hypertrophic cardiomyopathy and infiltrative disorders such as Fabry disease and amyloidosis. Both Fabry disease and amyloidosis are associated with proteinuria and peripheral neuropathy, but cardiac apical sparing favours amyloidosis. Friedreich ataxia is associated with hypertrophic cardiomyopathy and neuropathy, but heavy proteinuria is atypical. Given the nephrotic syndrome, absent myositis‐specific antibodies, and symptom progression on immunosuppression, we performed a kidney biopsy. Light microscopy indicated thickened glomerular basement membranes and mesangial expansion with eosinophilic material. Congo red stains were performed (Box 2), along with immunofluorescence studies and electron microscopy (Box 3), confirming a diagnosis of amyloidosis.

Box 2 **Kidney histology and Congo red staining**

**Figure:**
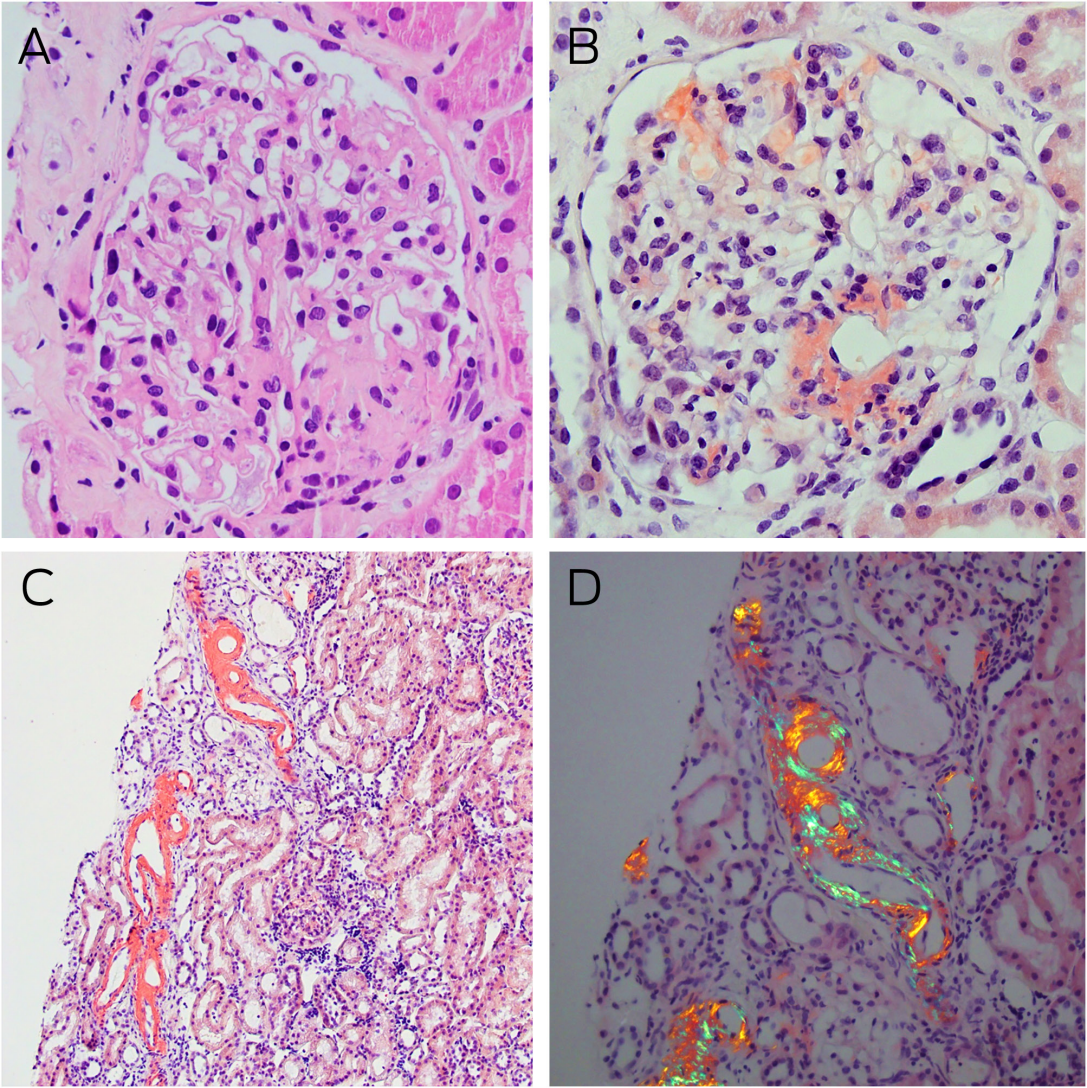
**A:** Light microscopy of a glomerulus with mildly globally thickened glomerular basement membranes, pale staining eosinophilic homogenous deposits in the capillary loops, mesangial areas, and hilar arterioles (haematoxylin–eosin stain, magnification × 400). **B:** Congo red staining showing typical salmon‐red deposits of amyloid in glomerular segments and around the hilum (magnification × 400). **C:** Similar Congo red positive staining is noted in some tubules in the renal cortex (magnification × 100). **D:** Congo red positive areas showing strong apple‐red/green birefringence under polarised light microscopy (magnification × 200).

Box 3 **Kidney immunofluorescence and electron microscopy**

**Figure:**
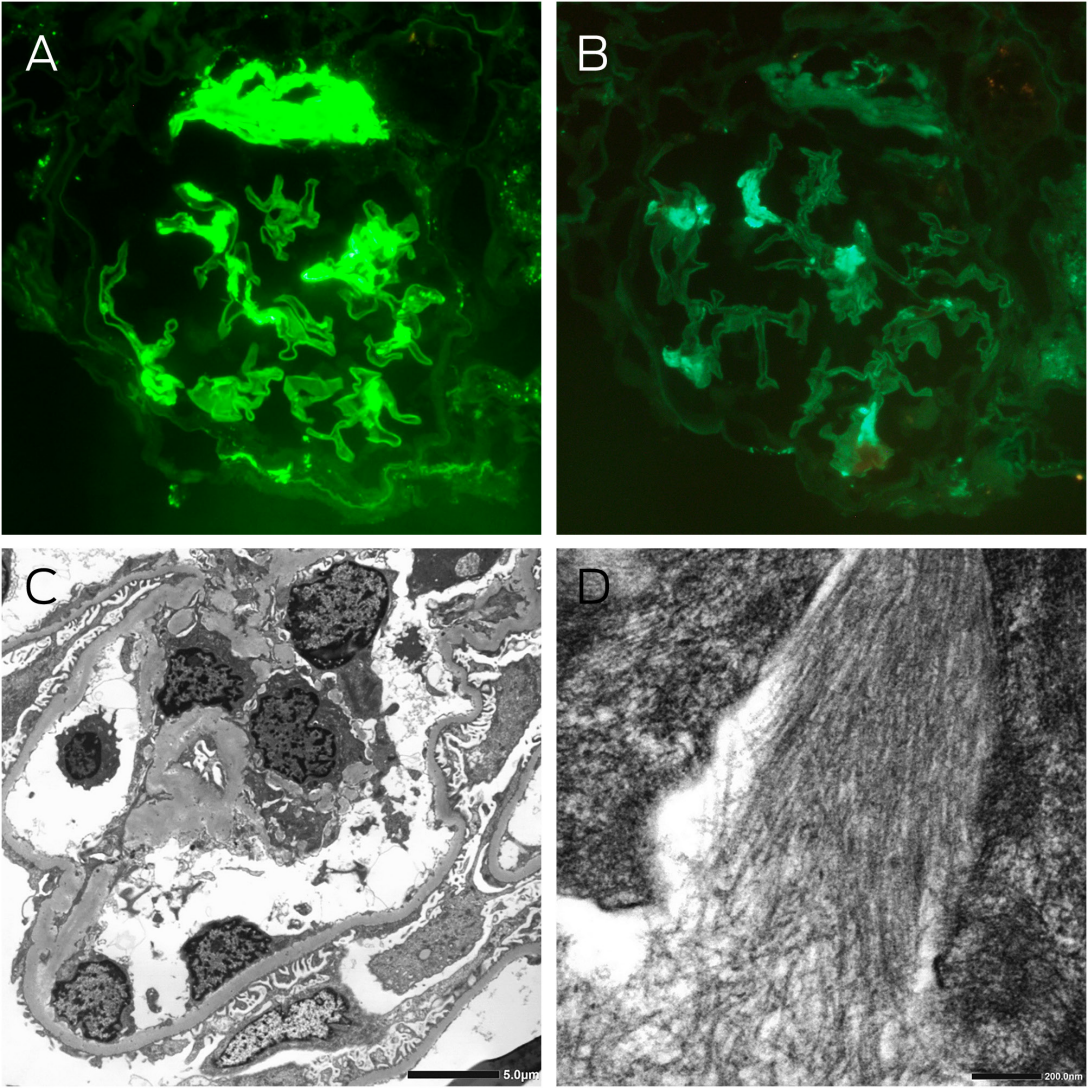
**A:** Immunofluorescence staining for λ light chains showing moderately intense segmental signal detection and strong signal detection at the hilum of a glomerulus (magnification × 400). **B:** Immunofluorescence staining for κ light chains showing light segmental signal detection in the glomerulus, which was significantly less than λ light chain detection (magnification × 400). **C:** Electron microscopy showing thickened glomerular basement membranes averaging 420 nm, with about 60% podocyte foot process effacement, and electron dense deposits in the capillary loops, mesangial and paramesangial areas. **D:** High power examination of these deposits revealed randomly oriented, straight, non‐branching fibrils with an average diameter of 11 nm.

Serum protein electrophoresis and free light chain (FLC) assays revealed an elevated λ‐FLC level of 470 mg/L (RI, < 26.3 mg/L) and normal κ‐FLC level of 16.6 mg/L (RI, < 19.4 mg/L), with a κ:λ ratio of 0.035 (RI, 0.26–1.65). Bone marrow biopsy showed a hypercellular marrow with 15% CD138+ plasma cells and Congo red positive deposits. The patient was treated with 13 cycles of bortezomib, cyclophosphamide and dexamethasone (VCD). After 3 months, his κ:λ FLC ratio normalised, and serum albumin level improved to 30 g/L with his urine protein–creatinine ratio falling to 0.07 g/mmol. He had repeat echocardiography 3 and 6 months after diagnosis, which showed persistent non‐dilated hypertrophied ventricles with severely reduced stroke volume, consistent with cardiac amyloidosis. His B‐type natriuretic peptide level was elevated 3 months after admission at 2112 ng/L (RI, < 100 ng/L), and he reported New York Heart Association class III symptoms of heart failure (comfortable at rest, but less than ordinary activity causes dyspnoea and fatigue).

## Discussion

We present a case of systemic light chain amyloidosis secondary to myeloma in a patient who initially presented with myopathy, heavy proteinuria, and peripheral oedema. Immunoglobulin light chain (AL) amyloidosis results from monoclonal FLC production from a clonal population of bone marrow plasma cells. Amyloid protein is insoluble and tissue deposition causes multiorgan dysfunction.^1^, ^2^ Diagnosis requires tissue samples of the involved organs, and biopsies of the heart, liver and kidney have high sensitivity (87–98%). A fat biopsy could be a less invasive option, but sensitivity depends on local expertise (80–93%).^2^ Although uncommon, AL amyloidosis can be diagnosed in muscle biopsies by deposits in blood vessels, connective tissue and encasing muscle fibres.^3^ Immunostaining for κ‐ and λ‐FLC is crucial for distinguishing AL amyloidosis from other subtypes but may not always be conclusive. Tandem mass spectrometry is the gold standard to determine amyloid type but is only available in certain centres, such as the national centre at Princess Alexandra Hospital in Brisbane.^4^

Renal involvement with AL amyloidosis typically manifests as nephrotic syndrome with variable renal impairment. Hepatomegaly and liver enzyme derangement (particularly cholestatic pattern) are common and may be confused with alcohol‐related effects. Neurological involvement such as painful peripheral neuropathy is less common. Cardiac signs and symptoms can be observed in 77% of patients.^5^ Cardiac involvement is associated with progressive cardiac failure with elevated B‐type natriuretic peptide levels, conduction disturbance or arrhythmia. Electrocardiograms may show low electrical voltages while transthoracic echocardiogram findings are of increased biventricular wall thickness with reduced diastolic velocities. Thus, amyloidosis should also be considered in cases of heart failure with preserved ejection fraction. Concentric remodelling and apical sparing resulting in a hyperdynamic apex is also characteristic, which generates the cherry‐on‐top pattern seen on strain maps of the left ventricle.^6^

Delayed diagnosis is common due to the non‐specific presentation of AL amyloidosis (median, 2.7 years from symptom onset).^5^ If myopathy is the presenting complaint, the median time to diagnosis is almost 2 years, and patients may be misdiagnosed with inflammatory myopathy and receive inappropriate treatment.^3^ In addition to VCD, daratumumab may improve haematological response but not mortality in cardiac amyloidosis. Select patients may be candidates for autologous stem cell transplantation (ASCT) and some centres are running trials of heart transplantation followed by ASCT. A clinical trial of doxycycline with bortezomib‐based treatment for cardiac AL amyloidosis is in progress (NCT03474458). The severity of cardiac involvement correlates with prognosis.^4^ For example, a prognostic staging system using cardiac biomarkers and the FLC difference (one point each for troponin T > 0.025 ng/mL, N‐terminal pro‐B‐type natriuretic peptide > 1800 pg/mL, FLC difference > 18mg/dL) indicated a median survival of 94 months with stage 1 (0 points) compared with 5.8 months with stage 4 disease (3 points).^6^

## Lessons from practice

- Muscle weakness is a rare presentation of amyloidosis but should be considered in the differential diagnosis of creatine kinase elevation without muscle‐specific antibodies.
- The findings of a non‐dilated cardiomyopathy, thickened ventricles with restrictive physiology and nephrotic syndrome are clinical clues of systemic amyloidosis.
- Once amyloidosis is diagnosed, a cause should be determined and a search for monoclonal protein and clonal disease should be conducted.
- Inform the pathologist that amyloidosis is a differential diagnosis so that a Congo red stain can be done on biopsy specimens.

## Open access

Open access publishing facilitated by Monash University, as part of the Wiley ‐ Monash University agreement via the Council of Australian University Librarians.

## Competing interests

No relevant disclosures.

## Provenance

Not commissioned; externally peer reviewed.
